# Job Satisfaction: Knowledge, Attitudes, and Practices Analysis in a Well-Educated Population

**DOI:** 10.3390/ijerph192114214

**Published:** 2022-10-31

**Authors:** Paolo Montuori, Michele Sorrentino, Pasquale Sarnacchiaro, Fabiana Di Duca, Alfonso Nardo, Bartolomeo Ferrante, Daniela D’Angelo, Salvatore Di Sarno, Francesca Pennino, Armando Masucci, Maria Triassi, Antonio Nardone

**Affiliations:** 1 Department of Public Health, University “Federico II”, Via Sergio Pansini N° 5, 80131 Naples, Italy; 2 Department of Law and Economics, University “Federico II”, 80131 Naples, Italy

**Keywords:** job satisfaction, knowledge, attitude, practice, cross-sectional survey

## Abstract

Job satisfaction has a huge impact on overall life quality involving social relationships, family connection and perceived health status, affecting job performances, work absenteeism and job turnover. Over the past decades, the attention towards it has grown constantly. The aim of this study is to analyze simultaneously knowledge, attitudes, and practices toward job satisfaction in a general population in a large metropolitan area. The data acquired from 1043 questionnaires—administered to subjects with an average age of 35.24 years—revealed that only 30% is satisfied by his job. Moreover, among all the tested sample, 12% receive, or often receive intimidation by their superior, and 23% wake up unhappy to go to work. Marital status and having children seem to be an important factor that negatively influences job satisfaction through worst behaviours. The multiple linear regression analysis shows how knowledge is negatively correlated to practices; although this correlation is not present in a simple linear regression showing a mediation role of attitudes in forming practices. On the contrary, attitudes, correlated both to knowledge and practices, greatly affect perceived satisfaction, leading us to target our proposed intervention toward mindfulness and to improve welfare regulation towards couples with children.

## 1. Introduction

Job satisfaction has been defined as a “pleasurable or positive emotional state, resulting from the appraisal of one’s job experiences” [1]. Job satisfaction reflects on overall life quality involving social relationships, family connection and perceived health status, affecting job performances, work absenteeism and job turnover, leading, in some cases, to serious psychological condition such as burnout [2,3,4,5,6].

The recent Gallup statistics on job satisfaction indicated that a very large portion of the world’s 1 billion full-time workers is disengaged, more precisely, only 15% of workers are happy and production in the workplace, the remaining 47% of workers are “not engaged,” psychologically unattached to their work and company [7]. In the EU, approximately one in five residents (16.9%) currently in employment expressed low levels of satisfaction with their job, on the other hand approximately one in four (24.6%) expressed high levels of satisfaction, the remaining residents (58.5%) declared medium levels of satisfaction with their job [8]. Characteristics such as age, sex, education, occupation, commuting time and difficulty as inadequate income, seems to be related to job satisfaction as they tent to influence expectation and preferences of individuals’ reflection on their perceived working condition [9,10]; however, as assessed in Eurofound, European Working Conditions Surveys [11] the relation between age and job satisfaction is very weak, although a slight increase in low satisfaction prevalence was found in elder population, it does not increase significantly with age even though expectations change during lifetime; educational attainment and income seem to play a significant role in job satisfaction as they grow in parallel, leading to better positions and a higher wages, along with power and more decisional autonomy. Sex is a factor as women seems to be overall more satisfied by their job in despite of the worst general conditions [11,12,13,14]. Job satisfaction also relates to marital status as single subjects’ results as the most satisfied by their work in some European Countries [15]. In Italy, the overall perceived job satisfaction seems to be similar to other regions in EU, and social relations as well as family composition appear to play a relevant role [16]. 

Job satisfaction has been studied mostly over a specific category of workers [17,18], as some types of works seems to be more related to pathological conditions such as burnout [19,20] and job-related stress [21,22,23]; however, as reported by those authors, this kind of selection method could lead to selection biases. According to van Saane [24], although many studies were carried as since Job Satisfaction broke out in the last 70’s as a central topic of interest, nor a mathematical instrument as reliable as desired nor a comparative method were found, usually those studies were based on single components of job satisfaction, taken out from extra working environment, and without analysing the consequences on behaviours in day life [25,26,27]. The literature research demonstrated that practices are the results of knowledge, attitudes, or their interaction. The KAP Survey Questionnaire [28] can be applied to highlight the main features of knowledge, attitude, and practice of a person, and to assess that person’s views on the matter. The purpose, when using the KAP Survey Model, is to measure a phenomenon through the quantitative collection method of a large amount of data through the administration of questionnaires and then statistically process the information obtained. Through a questionnaire, however, seems to be easier to quantify job satisfaction. In addition to that, studying broader populations’ consent to explore different components, both personal and environmental, which concur to influence it [29,30].

In the recent literature, a KAP model was used only once to analyse behaviours toward job satisfaction. In his work, Alavi [31] conducted a survey based cross-sectional study on 530 Iranian radiation workers; although it comprehends simultaneously knowledge, attitude, and practices, it was conducted on a specific category of workers and on a narrower population. Therefore, since to the best of our knowledge none of the studies presented in the literature are carried out on a broader population relating both knowledge and attitudes to behaviours on job satisfaction, the aim of this study is to analyse simultaneously knowledge, attitudes, and behaviours toward job satisfaction in a large metropolitan area. It is important to investigate this phenomenon to evaluate the condition and develop health education programs and community-based intervention to increase job satisfaction and knowledge and positively orienting attitudes.

## 2. Material and Methods 

### 2.1. Participants and Procedure

This cross-sectional study was conducted from November 2021 to February 2022 in the large metropolitan area of Naples, southern Italy, among working places, universities, and community centres. No specific category of participants was selected. In the questionnaire, respondents indicated their occupation by choosing from the following options: lawyer, architect, engineer, doctor, accountant, entrepreneur, teacher, law enforcement, trader, student, employee, worker, unemployed, other. Table 1 shows the categories indicated by the participants. The criteria for inclusion in the study required that respondents of a general population were over 18 years old, belonging to one of the categories of employment listed in Table 1, and resided in the metropolitan area of Naples. Every participant directly received a questionnaire (available upon request from the corresponding author) and at the time of filling out the questionnaire, the aim of the study and the anonymity and privacy of the data collecting method being used was explained, both in written form, as an introduction part of the questionnaire, and verbally to each of the participants. The questionnaire consisted of basic information about participants (age, gender, children, civil state, education level, profession, smoke habits) and three pools of questions divided in knowledge, attitudes and behaviours concerning their job satisfaction for a total number of 37 questions. The construction of the questionnaire was carried out as recommended by the KAP Model [28], briefly was divided into four phases: (1) Constructing the survey protocol; (2) Preparing the survey; (3) Course of the KAP survey in field; (4) Data analysis and presentation of the survey report. To develop the questionnaire, research questions based on the “Objectives of the study” were first carried out to develop the research questions, according to KAP Survey Model [28], the knowledge was considered as a set of understandings, knowledge, and “science” while Attitude as a way of being, a position. After, the research questions were reduced in number by removing those questions that require unnecessary information. When the above step is also done, the difficult questions have been changed/removed (closed questions have been used because one of the most important things that will increase the relevance of the questions is that the questions must be closed questions). Knowledge and attitudes were assessed on a three-point Likert scale with options for “agree”, “uncertain”, and “disagree”, while inquiries regarding behaviours were in a four-answer format of “never”, “sometimes”, “often”, and “yes/always”. A pilot study was also carried out to test the questionnaire and to verify the reliability of questions. Finally, all the collected questionnaires were digitalized submitting the codified answers in an Excel worksheet (MS Office).

### 2.2. Statistical Analysis

Data reported by the study were analysed using IBM SPSS (vers. 27) statistical software program. The analysis was carried out in two stages. In the first stage, a descriptive statistic was used to summarize the basic information of the statistical units. In the second stage, a Multiple Linear Regression Analysis (MLRA) was used to model the linear relationship between the independent variables and dependent variable.

The dependent variables (Knowledge, Attitudes and Behaviours) had been obtained by adding the scores obtained in the corresponding questions (questions with inverse answers have been coded inversely). The independent variables were included in all models: sex (1 = male, 2 = female); age, in years; education level (1 = primary school, 2 = middle school, 3 = high school, 4 = university degree); civil state (1 = Single; 2 = In a relationship; 3 = Married; 4 = Separated/Divorced; 5 = Widowed).

The main results from a MLRA contains the statistical significance of the regression model as well as the estimation and the statistical significance of the beta coefficients (*p*-value < 0.05) and the coefficient of determination (R-squared and adjusted R-squared), used to measure how much of the variation in outcome can be explained by the variation in the independent variables. Three MLRA were developed:

(1) Knowledge about job satisfaction (Model 1);
(2) Attitudes toward resilience and mindfulness (Model 2);
(3) Actual behaviours regarding Job and Job-related life (Model 3).

In Model 2, we added Knowledge to the independent variables, and in Model 3, we added Knowledge and Attitudes to the independent variables. In the analysis, we considered Attitudes and Knowledge as indexes rather than a scale, which means that each observed variable (A1, …, A13 and K1, …, K12) is assumed to cause the latent variables associated (Attitude and Knowledge). In other terms, the relationship between observed variables and latent variables is formative. Therefore, inter-observed variables correlations are not required. On the contrary, the relationship between the observed variables (B1, …, B14) and latent variable Behaviour could be considered reflective (Cronbach’s alpha = 0.825). All statistical tests were two-tailed, and the results were statistically significant if the *p*-values were less than or equal to 0.05.

## 3. Results and Discussion

Out of the 1057 participants, 1043 anonymous self-report surveys were returned, resulting in a response rate of 98.7%. Table 1 shows the characteristics of the study population: the mean age of the study population is 35.24 years; in 18–70 age range, the main group of distribution was 18–30 representing 44.6% of the sample; sex distribution shows that: 427 are men, 616 are woman. A large majority (73.5%) does not have children, while 26.5% of the sample has them. Most of the participants have a post graduate degree, while 29.1% are high school graduates. Among them, 22.2% are physicians, 15.1% teachers and 14.0% students (Table 1).

Respondent’s knowledge about job satisfaction is presented in Table 2. While a large majority of the sample population (91.7%) has a well-defined knowledge about job satisfaction main characteristics such as mains definitions, both of work-related stress and mobbing, most of them does not know or are not aware which risks are specifically related as only 31.4% knows that job related stress and mobbing are a threat to their cardiovascular health. Only 28.7% of the population knows that “Only 15% of worker, globally, are satisfied by their work” demonstrating that while knowledge regarding job related stress is well spread, the sample does not know how diffused it is and what kind of risks it involves, and that state provide a compensation for job related stress.

In Table 3 are described attitudes toward job satisfaction. Most of the participants think that working out is relaxing and spending time is regenerating, showing a good attitude to copy with work related stress. According to 93.4% of the sample, workload plays a key role in job satisfaction, as well as adequate wages and a clear task schedule. Several studies have enlightened that when workers lack a clear definition of the tasks which are necessary to fulfil a specific role, their levels of job satisfaction are likely to be negatively affected [32,33,34]. Interestingly, most of the population sees challenges as a motivation to do better (80.2%) and are motivated by career opportunities (90.7%); however, 50.5% of the population has a negative attitude about changes. In confirmation of that, when asked if “Changes lead to stress”, only a small fraction of the sample (14.6%) disagreed. This allowed us to assume that, although most of the population sees problems as an opportunity to learn, improve and progress in their work, they are aware of the difficulties connected to changing scenarios. About 27.2% of the sample does not have a positive attitude toward sharing their feeling about problems at work talking out loud. Bad interpersonal relationships with co-workers are another reason for job dissatisfaction. Poor or unsupportive relationships and conflicts with colleagues and/or supervisors lead to negative psychological intensions, resulting in job dissatisfaction [35,36].

Behaviours of respondents are listed in Table 4: A consistent part of the sample responded positively to the group of question toward behaviours regarding their coping level of stressful situation (B2, B4, B8, B9, B10) showing a reported good resilience. Commuting seems to be a problem for at least a third of the sample, also in a metropolitan area served by 2 subways, full bus service, car sharing services and a speedway. Job satisfaction is associated negatively with constraints such as commuting time. This dead time, mostly unpaid, is mandatory for workers to reach workplace. Although this is not considered as working time, and only a specific class is refunded, from the employers’ perspective, it is time dedicated to work and a strong determinant for low satisfaction levels. EU workers were much more likely to be highly (37.9%) or moderately satisfied (41.7%) with their commuting time compared to their job satisfaction. Most of the sample responded to not having experienced mobbing; although even a “low” result, such as a cumulative, summing both “yes/always” and “often”, of 11.8% is alarming and pushes us to study more about this phenomenon. Interestingly, 30.9% of respondents are satisfied about their work, reaching a total of 59.5%. In addition, with a “often” response showing a large appreciation of their jobs, 22.9% of the respondents “wake up unhappy to go to work”, and feel “stuck in a job with no career opportunities” (27.7%). The sample has no problems managing their work and social life (48.3%); however, only a complex of 35% of the sample usually spend their time with colleagues outside the office.

Table 5 illustrates results of linear multiple regression in three models: in Model I Knowledge, as dependent variable, correlate, with a *p*-value < 0.001; with “sex”, interestingly, woman seem to have a higher overall score of knowledge in disagreement with Gulavani [37] whose study was conducted among a sample of nurses and found no significant relation between sex and knowledge on job satisfaction. Al-Haroon [38] evidenced that among health workers, men had a better overall level of knowledge. These results, however, were collected over specific categories of employees, in a narrower sample; whereas our study was represented by a general population of a metropolitan area. No statistically significant correlation between knowledge and age, civil status, children, and education levels was encountered.

Previous research asses that attitude plays a key a role in job satisfaction, as some attitudinal characteristics of the subject influence perspective, coping skills and stressful situation management [39,40,41]. In Model II (Table 5) we correlated, through MLRA, attitudes with age, sex, civil state, having children, education, and overall knowledge score. With a *p*-value < 0.001, two correlations were found with education and overall knowledge score, both positively. Those results reflect, in accordance with Alavi [31], who found that higher level of education was among 3 factors that predicted job satisfaction and attaining a higher university degree compared to lower degrees contributes to a feeling of coherence, success at work, personal growth and self-respect, self-realization and intrinsic motivation, that education level and therefore a higher level of knowledge contributes to generating a sense of job satisfaction. In the questionnaire we tried to collect all those propension and as a result: in agreement with Hermanwan [42], Andrews [43] and Choi [44], subjects with better knowledge and high levels of education tent to have better attitudes.

In Model III, behaviours taken as a dependent variable are correlated to age, sex, civil state, children, education, knowledge, and attitudes. The results of linear multiple regression in this model assess that behaviours are negatively correlated to civil state, sons, and knowledge, and positively correlated to attitudes. Our findings show that there is a positive correlation between behaviours and attitudes, in agreement with previous literature [45,46,47], demonstrating that people with better attitudes tent to have a better overall behaviour. Surprisingly, in Model III, knowledge also has a statistically significant correlation to behaviours but in a negative way. This correlation, however, is not present when we correlate those variables alone in a Pearson’s correlation between knowledge, attitudes, and behaviours (Table 6). This evidence, therefore, suggests that attitude mediates the effect of knowledge on behaviours, assessing an important relation between those two determinants. People with a better overall score in behaviours tend to have a higher score in knowledge and attitude. In this sample, those who have a lower score in knowledge also has a higher behaviour score in accordance with a part of the previous literature [48,49]. This enlightens the importance of high levels of knowledge in order to form better attitudes in the pursuit of job satisfaction. Civil state and having children seem to play a key role in performing a better behaviour about job satisfaction; which is also evident in one specific question about behaviour: Question “B14” enlightens the social practices of subjects with colleagues outside the work environment, and the statistical analysis on this topic shows that subject with a more stable sentimental situation or with child tend to hang out with their colleagues less, likely worsening their relationships at work and getting a worse overall behaviour score and worse attitude toward the topic in agreement with Sousa-Poza [50] and Armstrong [51]. Job satisfaction has a strong correlation to family characteristics: Subjectst who have families with children have less positive behaviours towards their job satisfaction, directly affecting their overall behaviour score; this evidence is in contrast with Alavi [31], who states that job satisfaction is positively affected by family, assessing that “married employees have opportunities to receive support or advice from their family to mediate job conflicts,” Although he admits that in the literature, this result is controversial as some authors, such as Clark [52], found that “married employees experienced a higher level of job satisfaction than their unmarried co-workers”, and Booth and Van Ours’ [53], study did not find a statistically relevant correlation with the presence of children. Those results, therefore, suggest creating targeted educational programs, community-based intervention, and legal regulation, to improve self-awareness and resilience among workers, and a more practical intervention could be directed to families with child.

## 4. Conclusions

This study shows that the metropolitan population has general good knowledge about job satisfaction as well as a positive attitude. Job satisfaction, however, is reflected accordingly only with attitudes. While it has a negative relation to civil state and having children, this means that the experimental results of this study may be used to create targeted educational programs, community-based intervention, and legal regulation, to improve self-awareness and resilience among workers. A more direct intervention could be directed to families with children. Social networking with colleagues has an important impact on job satisfaction, as the part of the sample who responded positively to the specific question, had an overall better behaviour. Although, in this case, having children seems to be, as they negative correlate, a huge limitation to this practice. Considering that, as previously stated, the impact of job satisfaction on the population has a strong impact in terms of life balance, health, and economics, and it is well known that only a small fraction of workers are fully satisfied. It might be important to promote welfare regulation to allow a larger part of the population to conciliate work and family. Results of this paper could be an indicator of how to establish an educational program more efficiently. It is mandatory to strengthen specific knowledge about job satisfaction through the general population toward the importance of job satisfaction and the benefits related to a correct approach to work-life. The impact of a public health intervention could be even more effective by integrating another program to orient and define attitudes, which in turn will influence people to practice a mindfulness mental setting toward job satisfaction. In conclusion, a training program based on fundamental practices of job satisfaction should be improved in the young population, in early stage of family life, or before they have children, in order to achieve a double objective: “training family and spreading the practice to a future generation”.

## Figures and Tables

**Table 1:** Study population characteristics.

Study Population	N	Percentage
**Sex**	1043	
Male	427	40.9
Female	616	59.1
**Age**		
18–30	467	44.6
31–35	255	24.3
36–40	82	7.8
41–45	64	6.3
46–50	65	6.6
51–70	110	10.4
**Civil state**		
Single	298	28.6
Married	293	28.1
In a relationship	428	41.0
Divorced/Separated	15	1.4
Widowed	9	0.9
**Education**		
Middle school	38	3.6
Degree	681	65.3
Primary school	21	2.0
High school	303	29.1
**Profession**		
Architect	29	2.8
Business owner	29	2.8
Employee	158	15.1
Teacher	44	4.2
Dealer	19	1.8
Student	146	14.0
Others	189	35.6
Lawyer	76	7.3
Unemployed	10	1.0
Business Consultant	17	1.6
Physician	232	22.2
**Children**		
Yes	276	26.5
No	767	73.5

**Table 2:** Knowledge of respondents toward job satisfaction.

N.	Statement (*Variables*)	Agree (%)	Uncertain (%)	Disagree (%)
**K1**	Work related stress is more frequent in some professional categories.	86.5	6.7	6.8
**K2**	Men are more affected by work related stress than women.	6.5	21.4	72.1
**K3**	Work related stress is a condition that can be accompanied by physical, psychic, and social disturbs.	91.7	7.2	1.2
**K4**	Only 15% of worker, globally, are satisfied by their work.	28.7	61.6	9.7
**K5**	Worker from Northern Italy are more stressed than worker from Southern Italy.	16.1	34.0	49.9
**K6**	Mobbing is a form of physical and verbal abuse toward one or more people.	88.1	10.8	1.1
**K7**	Mobbing and work-related stress increase cardiovascular disease risk.	31.4	61.5	0.1
**K8**	Mobbing refers only to physical violence.	82.1	16.7	1.2
**K9**	Burnout is a syndrome linked to work related stress.	69.1	28.8	2.1
**K10**	INAIL * pays compensation from work related stress.	21.0	61.2	17.8

* INAIL: Istituto Nazionale Assicurazione Infortuni sul Lavoro (National Institute for Occupational Accident Insurance).

**Table 3:** Attitude of respondents toward job satisfaction.

N.	Statement (*Variables*)	Agree (%)	Uncertain (%)	Disagree (%)
**A1**	Workout is relaxing.	82.4	10.4	7.3
**A2**	Facing a problem there are multiple solutions.	77.6	19.4	3.1
**A3**	Facing an obstacle is demotivating.	18.9	31.6	49.5
**A4**	Challenges are a motivation to do better.	80.2	18.1	1.6
**A5**	Doing a work that satisfy us makes it easier.	88.3	6.7	5.0
**A6**	An inadequate wage makes work harder.	2.7	8.8	88.5
**A7**	Career opportunities push us to do better.	90.7	8.6	0.7
**A8**	Spending time outdoor is regenerating.	94.2	5.0	0.8
**A9**	Speaking openly of our work problem helps get through them.	72.9	21.5	5.7
**A10**	Changes lead to stress.	50.8	34.6	14.6
**A11**	Job related stress is underrated.	83.7	12.2	4.1
**A12**	An excessive workload can lead to job related stress.	93.4	5.9	0.7
**A13**	Unclear work tasks can cause stress.	86.1	12.3	1.6

**Table 4:** Behaviour of respondents toward job satisfaction.

N.	Questions	Yes (%)	Often (%)	Sometimes (%)	Never (%)
**B1**	Are you satisfied about your working life?	30.9	28.6	31.4	6.4
**B2**	Have you got troubles performing your daily duties?	6.0	10.7	61.8	21.4
**B3**	Do you manage to have a social life?	31.4	16.9	44.2	7.5
**B4**	Have you got trouble sleeping?	10.9	14.7	51.0	23.4
**B5**	Have you got trouble, with transportation, reaching your workplace?	20.5	11.2	34.3	33.9
**B6**	Do you drink alcohol after work?	4.4	6.0	37.5	52.1
**B7**	Do you receive pressions or intimidation from a superior?	5.9	5.9	33.6	54.6
**B8**	Do you think your workload is overwhelming?	12.8	17.5	50.3	19.4
**B9**	Have you got trouble focusing?	6.3	14.7	61.1	17.9
**B10**	Do you lose your temper if an unexpected event happens?	12.9	13.6	55.8	17.6
**B11**	Do you wake up unhappy to go to work?	11.4	11.5	55.8	21.3
**B12**	Have you got the feeling to be stuck in a job with no career opportunities?	17.6	10.1	37.4	34.9
**B13**	Do you skip work for health problems?	7.3	1.2	42.5	49.1
**B14**	Do you hang out with your colleagues outside the office?	18.0	17.0	47.5	17.5

**Table 5:** Results of the linear multiple regression.

	Coefficients Not Standardized		Coefficients Standardized		
	T	Standard Error	Beta	t	*p*-Value
**Model I—Dependent variable: Knowledge**					
Age	0.003	0.006	0.015	0.453	0.651
Sex	0.723	0.106	0.188	6.834	<0.001
Civil State	−0.011	0.058	−0.005	−0.181	0.857
Children	−0.043	0.138	−0.010	−0.313	0.754
Education	0.168	0.090	0.059	1.865	0.062
**Model II—Dependent variable: Attitudes**					
Age	−0.021	0.009	−0.070	−2.348	0.19
Sex	0.238	0.167	0.037	1.429	0.153
Civil State	0.076	0.090	0.021	0.842	0.400
Children	0.084	0.213	0.012	0.393	0.694
Education	1.433	0.132	0.300	10.831	<0.001
Knowledge	0.591	0.044	0.354	13.348	<0.001
**Model III—Dependent variable: Behaviour**					
Age	0.010	0.021	0.017	0.486	0.627
Sex	−0.771	0.398	−0.059	−1.940	0.053
Civil State	−0.742	0.213	−0.102	−3.475	<0.001
Children	−2.600	0.503	−0.177	−5.168	<0.001
Education	0.509	0.333	−0.052	1.530	0.126
Knowledge	−0.432	0.114	−0.126	−3.797	<0.001
Attitudes	0.537	0.072	0.262	7.427	<0.001

**Table 6:** Pearson’s correlation between knowledge, attitudes, and behaviours.

	Knowledge	Attitudes	Behaviours
**Knowledge**			
Pearson’s correlation	1	0.440	0.000
*p*-value		0.000	0.992
**Attitudes**			
Pearson’s correlation	0.440	1	0.248
*p*-value	0.000		0.000
**Behaviours**			
Pearson’s correlation	0.000	0.248	1
*p*-value	0.992	0.000	

## Data Availability

The data that support the findings of this study are available upon reasonable request from the corresponding author. The data are not publicly available due to privacy or ethical restrictions.
